# RNA-binding protein IGF2BP2 enhances circ_0000745 abundancy and promotes aggressiveness and stemness of ovarian cancer cells via the microRNA-3187-3p/ERBB4/PI3K/AKT axis

**DOI:** 10.1186/s13048-021-00917-7

**Published:** 2021-11-13

**Authors:** Shengtan Wang, Zaihong Li, Genhai Zhu, Lan Hong, Chunyan Hu, Kang Wang, Kaiying Cui, Chunbo Hao

**Affiliations:** 1 Department of Gynecology, Hainan General Hospital, Hainan Affiliated Hospital of Hainan Medical University, Haikou, 570011 Hainan PR China; 2 Department of Stomatology, Hainan General Hospital, Hainan Affiliated Hospital of Hainan Medical University, No. 19, Xiuhua Road, Haikou, 570011 Hainan PR China

**Keywords:** Ovarian cancer, IGF2BP2, circ_0000745, miR-3187-3p, ERBB4, PI3K/AKT

## Abstract

**Background:**

Circular RNAs (circRNAs) are increasingly recognized as important regulators in cancer including ovarian cancer (OC). This work focuses on the effects of circ_0000745 on the OC development of and molecules involved.

**Methods:**

Expression of circ_0000745 in collected OC tissues and the acquired OC cell lines was examined by RT-qPCR. The stability of circ_0000745 in cells was examined by RNase R treatment. The target transcripts interacted with circ_0000745 were predicted using bioinformatic systems. Gain- and loss-of-function studies of circ_0000745, microRNA (miR)-3187-3p and erb-b2 receptor tyrosine kinase 4 (ERBB4) were conducted to determine their functions on proliferation, migration, invasion and stem cell property of OC cells.

**Results:**

Circ_0000745 and ERBB4 were abundantly expressed while miR-3187-3p was poorly expressed in OC tissues and cells. Circ_0000745 sequestered miR-3187-3p and blocked its repressive effect on ERBB4. Downregulation of circ_0000745 reduced proliferation, aggressiveness, epithelial-mesenchymal transition, and stemness of SK-OV-3 cells, but this reduction was blocked upon miR-3187-3p inhibition or ERBB4 upregulation. By contrast, artificial induction of circ_0000745 upregulation, miR-3187-3p upregulation and ERBB4 downregulation led to inverse trends in ES-2 cells. ERBB4 promoted the phosphorylation of the PI3K/AKT signaling pathway. An RNA binding protein IGF2BP2 was found to circ_0000745 bind to and promote its expression and stability.

**Conclusion:**

This study demonstrated that circ_0000745 upregulated by IGF2BP2 promotes aggressiveness and stemness of OC cells through a miR-3187-3p/ERBB4/PI3K/AKT axis. Circ_0000745 may serve as a promising target for OC treatment.

## Introduction

Ovarian cancer (OC) represents one of the most frequent and life-threatening gynecologic tumors with high morbidity and mortality rates across the globe, with 295,414 new cases and 184,799 deaths reported in 2018 [1]. Surgical debulking and adjuvant chemotherapy combinations are the major option for OC management, though, the 5-year survival rate of advanced OC patients remained low at approximately 40% with only modest improvement in the past decades [2]. A major cause for the unfavorable prognosis is the limited access to an early diagnosis since patients are not frequently diagnosed until at advanced stages due to the lack of symptoms [3]. In addition, patients may acquire chemo-resistance due to the tumor recurrence and dissemination into surrounding organs, where the cancer stem cells are frequently involved [4]. Identifying biomarkers for OC early diagnosis and developing novel potential therapies are core issues for researchers in the field of OC management.

Circular RNAs (circRNAs) represent a major class of non-coding RNAs (ncRNAs) in animals in closed loop structure with potent regulatory ability and high stability [5]. These ubiquitous, stable, and conserved RNA molecules with an array of activities including translation and splicing regulation, are capable of interacting with RNA binding proteins (RBPs) and microRNA (miRNA) [6]. Due to the versatile functions, they have been increasingly recognized to be involved in the pathogenesis of multiple cancer types, including OC [6]. Circ_0000745 is a circRNA spliced from the sperm antigen with calponin homology and coiled-coil domains 1 (SPECC1), located on chromosome 17, which has been reported to promoter tumorigenesis of cervical cancer [7]. But its functions in OC are unexplored. One well-established regulatory network of circRNAs is that they may sequester miRNAs to block the suppressive function of miRNAs on other transcripts, termed competing endogenous RNAs (ceRNAs) [8]. In this paper, our bioinformatic analyses predicted a potential circ_0000745/miR-3187-3p/erb-b2 receptor tyrosine kinase 4 (ERBB4) axis in OC. Poor expression of miR-3187-3p has been reported to be associated with reduced recurrence-free survival rate in patients with bladder cancer [9]. ERBB4 is a member of the epidermal growth factor receptor (EGFR) family which are crucial regulators in the tumorigenesis [10]. Importantly, suppression of ERBB4 by miRNAs binding at the 3’untranslated region (3’UTR) has been demonstrated to lead to better prognosis of patients with OC [11]. In addition, RBPs have been reported to interact with circRNAs and enhance their stability and abundancy[12]. Here, the bioinformatic analyses suggested insulin like growth factor 2 mRNA binding protein 2 (IGF2BP2) as a highly expressed RBP in OC that can bind to curc_0000745. Taken together, we hypothesized that IGF2BP2 enhances circ_0000745 abundancy and promotes OC progression through a miR-3187-3p/ERBB4 axis.

## Materials and methods

### Collection of clinical samples

Fifty OC patients treated at Hainan General Hospital, Hainan Affiliated Hospital of Hainan Medical University from February 2017 to May 2019 were recruited in this study. All patients were free of a history of radio- or chemo-therapy and without any other malignancies. The tumor tissues and the para-cancerous tissue of patients were collected during surgery and immediately frozen at − 80 °C until subsequent use. This study was ratified by the Ethics Committee of Hainan General Hospital, Hainan Affiliated Hospital of Hainan Medical University (2017–01-06) and performed as per the *Declaration of Helsinki*. Written informed consent form was received from each enrolled subject.

### Cell incubation and transfection

A human epithelial ovarian cell line IOSE-80 (MZ-2207) was purchased from Ningbo Mingzhou Biotechnology Co., Ltd. (Zhejiang, China). The OC cell lines CoC1 (CL-0064), ES-2 (CL-0079), SW626 (CL-0226) and SK-OV-3 (CL-0215) were acquired from Procell Life Science & Technology (Wuhan, Hubei, China) and underwent short tandem repeat loci examination. All cells were incubated in Roswell Park Memorial Institute-1640 (Gibco Company, Grand Island, NY, USA) containing 10% fetal bovine serum (FBS, Gibco) and 1% penicillin/streptomycin (Sigma-Aldrich Chemical Company, St Louis, MO, USA) at 37 °C with 5% CO_2_.

The short hairpin (sh) RNAs (sh-circ 1, 2, 3#, sh-ERBB4 1, 2, 3# and sh-IGF2BP2 1, 2, 3#), pCE-RB-Mam vector-based overexpression (oe) vectors of circ_0000745 (oe-circ), pEXP-RB-Mam-based vector of ERBB4 and IGF2BP2 (oe-ERBB4 and oe-IGF2BP2), and the control vectors were purchased from Guangzhou RiboBio Co., Ltd. (Guangdong, China). The miR-3187-3p mimic, miR-3187-3p inhibitor and the controls were purchased from GenePharma Co., Ltd. (Shanghai, China). All transfections were conducted using a Lipofectamine™ 2000 kit (Thermo Fisher Scientific Inc., Waltham, MA, USA) according to the manufacturer’s protocol.

### Reverse transcription quantitative polymerase chain reaction (RT-qPCR)

Total RNA was extracted using an RNeasy Mini Kit (Qiagen, Valencia, CA, USA). For circRNA and mRNA, the RNA was reverse-transcribed into cDNA using a Prime Script RT Master Mix (TaKaRa Biotechnology Ltd., Dalian, China). Next, qPCR was conducted using SYBR Green Master Mix (TaKaRa) and PCR LightCycler480 (Roche Diagnostics, Basel, Switzerland), and glyceraldehyde-3-phosphate dehydrogenase (GAPDH) was used as control. For miRNA, cDNA was synthesized using a PrimeScript™RT kit (TaKaRa), and the expression of miR-3187-3p was examined using the TaqMan Universal Master Mix II (Applied Biosystems, Inc., Carlsbad, CA, USA) with U6 as the internal loading. The primer sequences are listed in Table 1.

**Table 1:** Primer sequences for RT-qPCR

Gene	Primer sequence (5′-3′)
circ_0000745	F: ATGTTGAAAGTAGCCCGAGCAG
	R: TGGGAGTGTTGGAAGAAGTTGG
SPECC1	F: ACCCCAGGAAATCAGTGTCCA
	R: GTTCCCGAACTTGGGACTCAA
miR-3187-3p	F: CCTGGGCAGCGTGTGGCT
	R: CTCAACTGGTGTCGTGGA
ERBB4	F: GGAGTATGTCCACGAGCACAAG
	R: CGAGTCGTCTTTCTTCCAGGTAC
IGF2BP2	F: GTTGGTGCCATCATCGGAAAGG
	R: TGGATGGTGACAGGCTTCTCTG
GAPDH	F: GGGAAACTGTGGCGTGAT
	R: GAGTGGGTGTCGCTGTTGA
U6	F: CTCGCTTCGGCAGCACA
	R: AACGCTTCACGAATTTGCGT

*Note*: *RT-qPCR* Reverse transcription quantitative polymerase chain reaction, *SPECC1* Sperm antigen with calponin homology and coiled-coil domains 1, *miR* microRNA, *ERBB4* Erb-b2 receptor tyrosine kinase 4, IGF2BP2, insulin like growth factor 2 mRNA binding protein 2, *GAPDH* Glyceraldehyde-3-phosphate dehydrogenase, *F* Forward, *R* Reverse

### RNase R treatment

Total RNA was detached in RNase R (40 U, TianGen Biotech Co., Ltd., Beijing, China) at 37 °C for 15 min. Then, an equal volume of RNA was reverse-transcribed into cDNA. The circ_0000745 expression in this setting was analyzed by RT-qPCR again. The expression level was considered to be positively linked to the stability of circ_0000745 and its linear counterpart, SPECC1 mRNA.

### Western blot analysis

Total protein was extracted using the radio-immunoprecipitation assay cell lysis buffer (Beyotime Biotechnology Co., Ltd., Shanghai, China) containing total protein. After concentration determination, an equal volume of protein was separated by 10% sodium dodecyl sulfate-polyacrylamide gel electrophoresis and transferred onto polyvinylidene fluoride membranes (Millipore Corp., Billerica, MA, USA). The membranes were blocked by 5% non-fat milk and co-cultured with the primary antibodies against E-cadherin (1:1000, #3195, Cell Signaling Technology (CST), Beverly, MA, USA), Vimentin (1:1000, #5741, CST), Snail (1:1000, #3879, CST), GAPDH (1:1000, #5174, CST), p-protein kinase B (p-AKT1, phospho S473) (1:1000, ab81283, Abcam Inc., Cambridge, MA, USA), p-phosphatidyl inositol 3-kinase (p-PI3Kinase p85 alpha, 1:1000, ab191606, Abcam) and IGF2BP2 (1:1000, ab129701, Abcam) at 4 °C overnight, and then with secondary antibody IgG H&L (HRP) (1:10000, ab205718, Abcam) at 20 °C for 2 h. The protein blots were visualized using the Immobilon ECL substrate (Millipore), and the images were captured using an Optimax X film processor. Relative protein expression was examined using Image J (NIH), and GAPDH was used for internal loading.

### Cell counting Kit-8 (CCK-8) method

A CCK-8 kit (Beyotime) was applied to explore proliferation of cells. In short, transfected cells were cultured in 96-well plates at 1000 cells per well. At 0, 24, 48, 72 h, respectively, each well was loaded with 10 μL CCK-8 solution, followed by another 2 h of incubation at 37 °C. The optical density (OD) value at 450 nm was read using a spectrophotometer (Yidian Analytical Instrument Co., Ltd., Shanghai, China).

### Colony formation assay

Transfected cells were incubated in 6-well plates at 3000 cells per well for 2 weeks of normal incubation. Thereafter, the cells were washed in phosphate-buffered saline (PBS), fixed in 4% paraformaldehyde for 15 min, and then stained with 0.1% crystal violet for 30 min. The number of colonies formed by cancer cells was observed and calculated under the microscope.

### Wound healing assay

After transfection, 1 × 10^6^ OC cells were cultured in 6-well plates till cell adherence. Then, a sterile 200-μL pipette tip was utilized to produce scratches on the cells. Next, the scratched cells were removed, and the remaining cells were incubated in serum-free medium. The width of the scratches at the 0 and 48 h was photographed and observed. The migration of cells in 48 h was measured according to the ratio of 48-h width to 0-h width using the Image J software.

### Transwell assay

The Transwell assay was further performed to analyze the invasion ability of cells. In short, 2 × 10^4^ transfected OC cells were resuspended in 200 μL serum-free medium. The cells were sorted in apical chambers with 8-μm pores and pre-coated with Matrigel (BD Biosciences, NJ, USA). The basolateral chambers were loaded with 300 μL 10% FBS-contained medium. After 24 h, the invaded cells were fixed and stained with 0.1% crystal violet for 1 h. The number of invaded cells was calculated under the microscope (Olympus Optical Co., Ltd., Tokyo, Japan).

### Sphere formation assay

The stem cell property of OC cells was examined using a sphere formation assay as per a previous report [13]. In short, cells were resuspended in DMEM/F12 medium (1:1) which was supplemented with 1% FBS, 1% penicillin/streptomycin, 10 ng/mL recombinant basic fibroblast growth factor and 20 ng/mL recombinant epidermal growth factor, and cultured in 6-well ultra-low attachment plates (Corning, NY, USA). After 14 d, the number of formed spheres was counted under the microscope.

### Nuclear-cytoplasmic RNA separation assay

The nuclear and cytoplasmic RNA of OC cells was extracted using a PARIS™ Kit (Thermo Fisher Scientific) in line with the kit’s protocols. Relative expression of circ_000745 in cytoplasm and in nucleus was examined using RT-qPCR. GAPDH was used as the internal control for cytoplasm while U6 for nucleus.

### Dual-luciferase reporter gene assays

Putative binding sites between circ_0000745 or ERBB4 3′-UTR and miR-3187-3p, termed wild-type (WT) sequences were obtained from StarBase (http://starbase.sysu.edu.cn/), and the mutant-type (MT) sequences were designed as well. The sequences were inserted into pGL3 promoter plasmids (Promega, Fitchburg, WI, USA) to construct circ-WT and ERBB4-WT or circ-MT and ERBB4-MT luciferase reporter plasmids. These plasmids were co-administrated with miR-3187-3p mimic or negative control (NC) mimic into 293 T cells. After 48 h of warm incubation, the cells were harvested, and the relative luciferase activity in cells was examined using a dual-luciferase reporter assay system (Promega).

### RNA pull-down assay

Circ_0000745 was labeled by biotin (Bio-circ), and Bio-NC served as the control. OC cells were lysed in lysis buffer containing RNase inhibitor. Next, the Bio-circ or Bio-NC was incubated with the supernatant of the lysis buffer at 4 °C for 4–6 h. The reaction solution was further loaded with streptomycin beads for 1–2 h at 20 °C. After that, the magnet beads were washed 2–3 times, and the expression of IGF2BP2 protein in the complexes was examined by western blot analysis.

### Xenograft tumors in nude mice

Animal experiments were approved by the Animal Ethics Committee of Hainan General Hospital, Hainan Affiliated Hospital of Hainan Medical University (2021–09-01) and performed according to the Guide for the Care and Use of Laboratory Animals (NIH Publication No. 85–23, revised 1996). Twenty four-week-old BALB/c nude mice procured from Vital River Laboratory Animal Technology Co., Ltd. (Beijing, China) were allocated into four groups (sh-NC, sh-IGF2BP2, sh-IGF2BP2 + oe-NC and sh-IGF2BP2 + oe-circ), *n* = 5 in each group.

Stably transfected SK-OV-3 cells were injected into the right flank of mice (5 × 10^6^ cells per mouse) via subcutaneous injection. The volume (V) of the xenograft tumors was examined every 7 d as follows: V = 0.5 × a × b^2^, where “a” indicates the long axis and “b” indicates the short axis. After 5 weeks, the animals were sacrificed via intraperitoneal injection of 120 mg/kg pentobarbital sodium. The tumor tissues were collected and weighed and used for subsequent examinations.

### Immunohistochemistry (IHC)

The collected xenograft tumor tissues were fixed in 10% formaldehyde, embedded in paraffin, cut into sections, dewaxed, and rehydrated. Then, the sections were treated with 0.01 M citrate buffer for antigen retrieval, incubated with H_2_O_2_ solution (3%) at room temperature for 15 min, and incubated with goat serum (5%) for 30 min. Thereafter, the sections were incubated with the primary antibodies against IGF2BP2 (1:100, ab129071, Abcam) or ERBB4 (1:1000, ab219208, Abcam) at 4 °C overnight, and then with goat anti-rabbit IgG H&L (HRP) (1:2000, ab205718, Abcam) at room temperature for 30 min. After DAB and hematoxylin staining, the sections were sealed with neutral resin and observed under the microscope and analyzed using Image J.

### Statistical analysis

Data were analyzed using Prism 8.0 (GraphPad, La Jolla, CA, USA). All data were exhibited as the mean ± standard deviation (SD) from three repetitions. Differences were analyzed by the *t* test (two groups) and one-way or two-way analysis of variance (ANOVA) followed by Turkey’s post hoc test (multiple groups). Clinicopathological features of patients were analyzed by the Fisher’s exact test or Chi-square test. Correlations between variables were measured by the Pearson’s correlation analysis. *p < 0.05 represents significant difference.

## Results

### Circ_0000745 is abundantly expressed OC tissues and cells

**Fig. 1:**
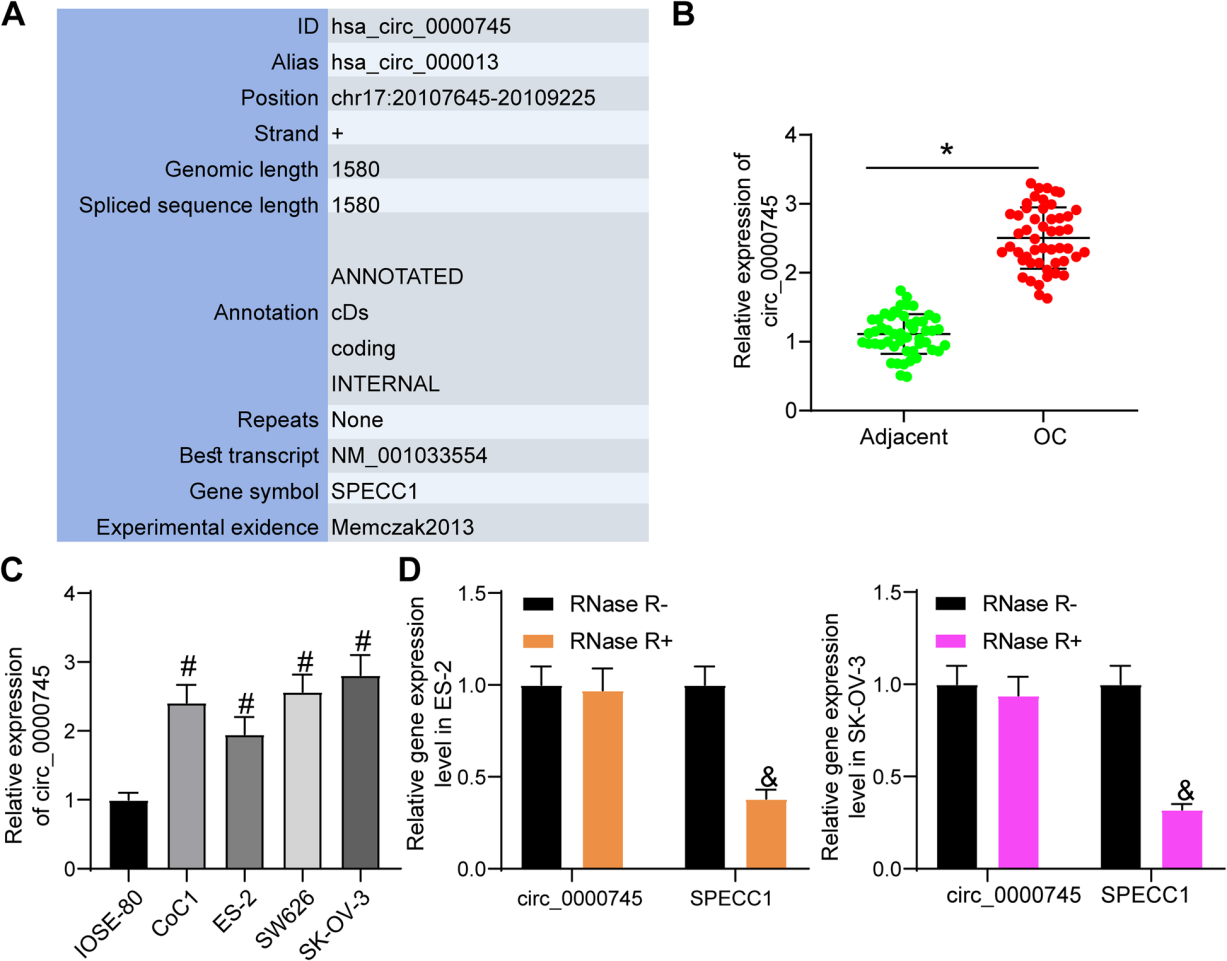
Circ_0000745 is abundantly expressed OC tissues and cells. **A**, sequence information of circ_0000745; **B**, expression of circ_0000745 in OC tumor tissues and the paired para-cancerous tissues examined by RT-qPCR; **C**, expression of circ_0000745 in the OC cell lines (CoC1, ES-2, SW626 and SK-OV-3) and in normal epithelial ovarian cells (IOSE-80) quantified by RT-qPCR; **D**, stability of circ_0000745 in OC cells examined by RNase R treatment. Data were exhibited as mean ± SD from three repetitions. Differences were analyzed by paired *t* test (**B**), one-way ANOVA (**C**) or two-way ANOVA (**D**); *p < 0.05 vs. adjacent; #*p* < 0.05 vs. IOSE-80 cells; & *p* < 0.05 vs. RNase-R

We first obtained the sequence information of circ_0000745 (chr17:20107645–20,109,225) from the CircBase system (http://circbase.org/), which suggested that it is trans-spliced from a linear mRNA SPECC1. The sequence information of circ_0109046 was obtained from CircBase (Circbase.org/) (Fig. 1A). Next, the expression of circ_0000745 in the collected tissue samples from OC patients was examined using RT-qPCR. It was found that the circ_0000745 expression was increased in OC tumor tissues compared to the adjacent tissues (Fig. 1B). According to the mean value of circ_0000745 expression (2.51), the patients were allocated into low (*n* = 26) and high (*n* = 24) circ_0000745 expression groups. Analyses on the clinicopathological features of patients suggested that high circ_0000745 expression was correlated with advanced FIGO stage and pathological grades (Table 2).

**Table 2:** Associations of circ_0000745 expression with the clinicopathological features of patients with OC

Clinicopathological features		circ_0000745 expression		*P* value
		Low (*n* = 26)	High (*n* = 24)	
Age (years)	≥50 (*n* = 17)	10	7	0.5592
	< 50 (*n* = 33)	16	17	
Histological type	Epithelial tumor (*n* = 35)	15	20	0.1297
	Germ cell tumor (*n* = 9)	7	2	
	Other tumors (*n* = 6)	4	2	
FIGO stage	I-II (*n* = 21)	16	5	**0.0047
	III-IV (*n* = 29)	10	19	
Pathological grade	G1-G2 (*n* = 23)	16	7	*0.0269
	G3 (*n* = 27)	10	17	

*Note*: Clinicopathological features of patients were analyzed by the Fisher’s exact test or Chi-square test; *OC* Ovarian cancer, *FIGO* Federation of Gynecology and Obstetrics

In the subsequent cellular experiments, RT-qPCR also confirmed higher expression of circ_0000745 in the OC cell lines (CoC1, ES-2, SW626 and SK-OV-3) than that in IOSE-80 cells (Fig. 1C). Among the OC cell lines, the SK-OV-3 cell line with the highest expression of circ_0000745, and the ES-2 cell line with the lowest expression of circ_0000745, were selected for subsequent use.

To examine the stability of circ_0000745 in OC cells, total RNA extracted from SK-OV-3 cells and ES-2 cells was detached in RNase R (RNase R+) or not (RNase R-). The expression of circ_0000745 and the linear mRNA SPECC1 in cells relative to the internal control in the RNase R- group was examined using RT-qPCR (Fig. 1D). It was found that RNase R treatment led to a significant degradation of mRNA SPECC1, but it had no impact on circ_0000745, which validated the circular structure and stability of circ_0000745 in cells.

### Cir0063_0000745 downregulation suppresses aggressiveness and stemness of OC cells

**Fig. 2:**
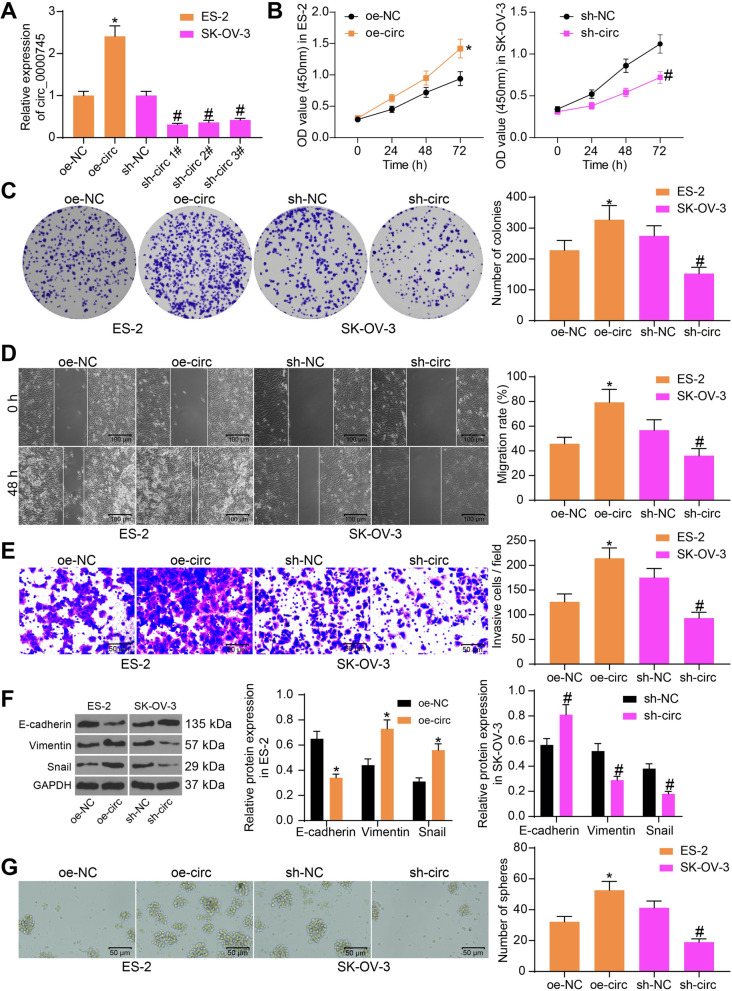
Circ_0000745 downregulation suppresses aggressiveness and stemness of OC cells. **A**, transfection efficacy of oe-circ and shRNAs in ES-2 or SK-OV-3 cells examined by RT-qPCR; **B**, proliferation of ES-2 and SK-OV-3 cells examined by the CCK-8 assay; **C**, colony formation ability of ES-2 and SK-OV-3 cells detected by the colony formation assay; **D**, migration ability of ES-2 and SK-OV-3 cells examined by the wound-healing assay; **E**, invasion ability of ES-2 and SK-OV-3 cells detected by the Transwell assay; **F**, protein levels of EMT-related markers in ES-2 and SK-OV-3 cells quantified by western blot assay; **G**, stem cell property of ES-2 and SK-OV-3 cells examined by the sphere formation assay. Data were exhibited as mean ± SD from three repetitions. Differences were analyzed by one-way ANOVA (**A**, **C**, **D**, **E** and **G**) or two-way ANOVA (**B** and **F**); *p < 0.05 vs. oe-NC; #*p* < 0.05 vs. sh-NC

To confirm the relevance of circ_0000745 to the behaviors of OC cells, oe-circ was transfected into ES-2 cells for circ_0000745 upregulation, while shRNAs (sh-circ 1, 2, 3#) were administrated into SK-OV-3 cells for circ_0000745 knockdown. Successful transfections of oe-circ or shRNAs were confirmed by RT-qPCR (Fig. 2A), and sh-circ 1# with the best interfering efficacy was selected for subsequent use.

Next, proliferation of cells was examined. According to the CCK-8 assay, overexpression of circ_0000745 enhanced the proliferation of ES-2 cells, whereas downregulation of circ_0000745 in SK-OV-3 cells reduced cell proliferation (Fig. 2B). Similar trends were found by the colony formation assay where we found increased colonies formed by ES-2 cells while decreased colonies formed by SK-OV-3 cells (Fig. 2C). In addition, the aggressiveness of OC cells in the setting of circ_0000745 upregulation or downregulation was examined as well. According to the wound healing and Transwell assays, upregulation of circ_0000745 enhanced the migratory (Fig. 2D) and invasive (Fig. 2E) potentials of ES-2 cells; still the migration and invasion of SK-OV-3 cells was suppressed upon circ_0000745 silencing. The levels of epithelial-mesenchymal transition (EMT)-related marker proteins in cells were quantified by western blot analysis. It was found that oe-circ enhanced the expression of mesenchymal marker proteins Vimentin and Snail but reduced the expression of the epithelial marker E-cadherin in ES-2 cells (Fig. 2F). Again, inverse trends were found in SK-OV-3 cells where circ_0000745 was suppressed. The stemness of OC cells was examined by the sphere formation assay. The number of tumor spheres formed by ES-2 cells was increased whereas that by SK-OV-3 cells was reduced (Fig. 2G). These results indicated that silencing of circ_0000745 suppressed the stem cell property of OC cells as well.

### Circ_0000745 directly binds to miR-3187-3p

**Fig. 3:**
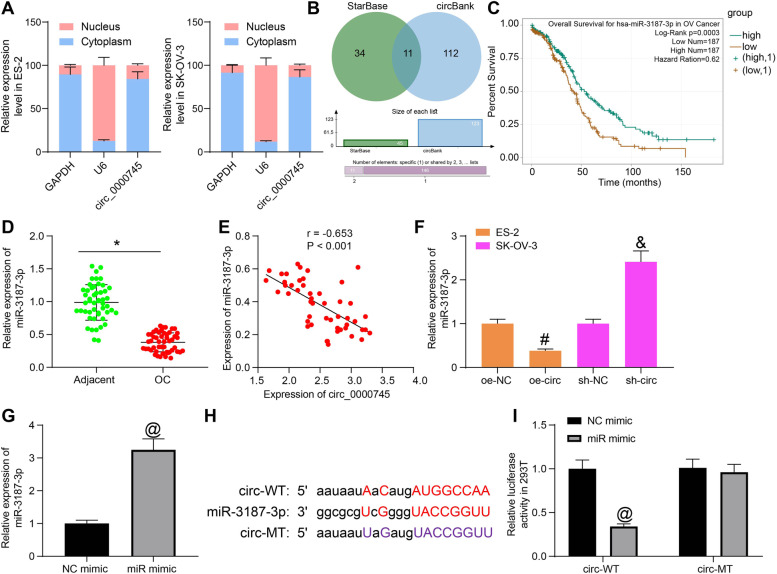
Circ_0000745 directly binds to miR-3187-3p in OC cells. **A**, sub-cellular localization of circ_0000745 in OC cells examined by a nuclear-cytoplasmic RNA separation assay; **B**, intersections of the candidate target miRNAs of circ_0000745 predicted using StarBase and CircBank; **C**, correlations between the candidate miRNAs and the survival rate of OC patients predicted on the Pan-cancer platform on StarBase; **D**, miR-3187-3p expression in the collected tissue samples detected by RT-qPCR; **E**, a negative correlation between the expression of miR-3187-3p and circ_0000745 in OC tissues; **F**, expression of miR-3187-3p in ES-2 and SK-OV-3 cells after oe-circ or sh-circ transfection determined by RT-qPCR; **G**, transfection efficacy of miR-mimic in 293 T cells confirmed by RT-qPCR; **H**, putative binding site between miR-3187-3p and circ_0000745 and the MT sequence for luciferase assay; **I**, luciferase activity of circ-WT and circ-MT in 293 T cells examined by the luciferase reporter gene assay. Data were exhibited as mean ± SD from three repetitions. Differences were analyzed by paired *t* test (**D**), unpaired *t* test (**G**), one-way ANOVA (**F**) or two-way ANOVA (**I**); *p < 0.05 vs. adjacent; @ *p* < 0.05 vs. NC mimic; #*p* < 0.05 vs. oe-NC; &*p* < 0.05 vs. sh-NC; In panel **E**, the correlation between miR-3187-3p and circ_0000745 was validated using Pearson’s correlation analysis, *r* = − 0.653, *p* < 0.001

To explore the potential mechanism involved, we first examined the sub-cellular localization of circ_0000745 in OC cell lines. The nuclear-cytoplasmic RNA separation assay suggested that circ_0000745 was mainly distributed in cytoplasm (Fig. 3A). Next, we predicted the candidate target miRNAs of circ_0000745 using bioinformatic systems, including StarBase and CircBank (http://www.circbank.cn/), and 11 common outcomes were predicted (Fig. 3B). We then analyzed the correlations between these candidate miRNAs and the survival rate of OC patients using the Pan-cancer platform on StarBase. Only miR-3187-3p showed a strong relevance to the survival of patients. Patients with higher miR-3187-3p abundancy were suggested to have better survival opportunity (Fig. 3C). Next, we explored the expression of miR-3187-3p in the collected tissue samples. Consequently, miR-3187-3p was found to be poorly expressed in the tumor tissues (Fig. 3D), which showed a converse correlation with circ_0000745 (Fig. 3E).

The expression of miR-3187-3p in ES-2 and SK-OV-3 cells was then examined. It was found that oe-circ in ES-2 cells reduced the expression of miR-3187-3p, whereas sh-circ in SK-OV-3 cells elevated the miR-3187-3p abundancy (Fig. 3F). Next, the miR-3187-3p mimic or the NC mimic was transfected into 293 T cells, and the successful transfection was confirmed by RT-qPCR (Fig. 3G). We then obtained the putative binding site between circ_0000745 and miR-3187-3p from StarBase and designed the MT binding sequence for luciferase assay (Fig. 3H). The subsequent luciferase assay suggested that miR mimic led to a notable decline in the luciferase activity of circ-WT vector in 293 T cells, but the luciferase activity of circ-MT in cells was not changed (Fig. 3I), which indicated a direct binding relationship between circ_0000745 and miR-3187-3p.

### miR-3187-3p blocks the functions of circ_0000745 in OC cells

**Fig. 4:**
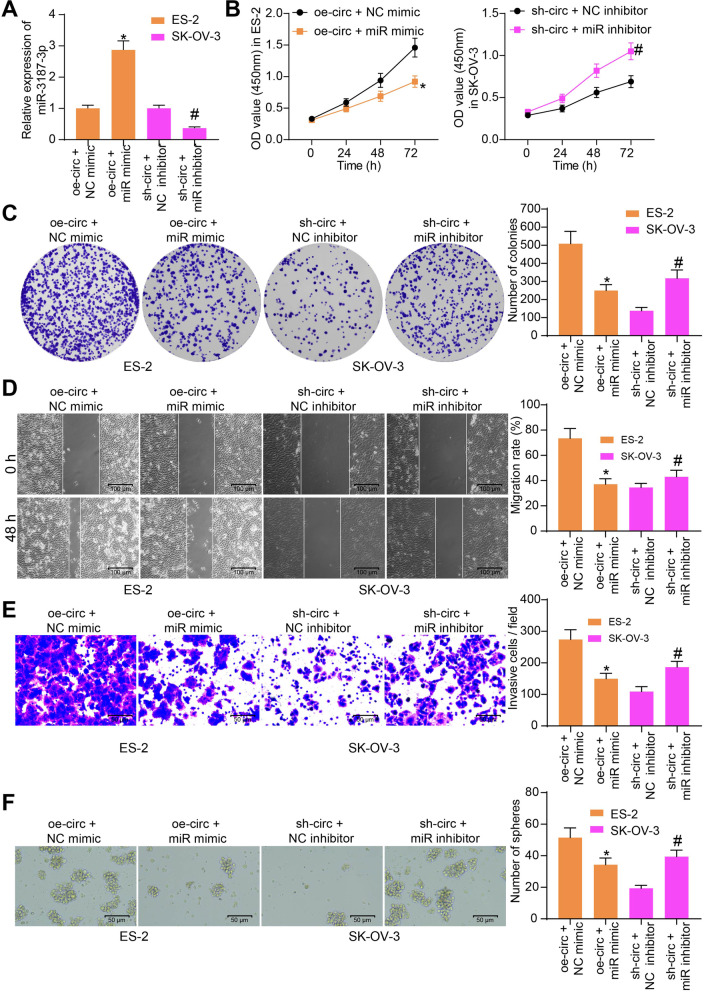
miR-3187-3p blocks the functions of circ_0000745 on OC cells. **A**, transfection efficacy of miR mimic/inhibitor examined by RT-qPCR; **B**, proliferation ability of ES-2 and SK-OV-3 cells examined by the CCK-8 assay; **C**, colony formation ability of ES-2 and SK-OV-3 cells measured by the colony formation assay; **D**, migration ability of ES-2 and SK-OV-3 cells examined by the wound-healing assay; **E**, invasion ability of ES-2 and SK-OV-3 cells determined by the Transwell assay; **F**, stem cell property of ES-2 and SK-OV-3 cells examined by the sphere formation assay. Data were exhibited as mean ± SD from three repetitions. Differences were analyzed by one-way ANOVA (**A**, **C**, **D**, **E** and **F**) or two-way ANOVA (**B**); *p < 0.05 vs. oe-circ + NC mimic; #*p* < 0.05 vs. sh-circ + NC inhibitor

To further confirm the function of miR-3187-3p on OC cells, rescue experiments were conducted through administration of miR mimic in ES-2 cells and miR inhibitor in SK-OV-3 cells on the basis of oe-circ or sh-circ transfection, and the transfection efficacy was examined by RT-qPCR again (Fig. 4A). Under these conditions, the CCK-8 assay suggested that the proliferation of ES-2 cells enhanced by circ_0000745 was blocked by miR-3187-3p, whereas miR-3187-3p inhibition restored the proliferation of SK-OV-3 cells (Fig. 4B). Likewise, we confirmed that miR-3187-3p blocked the colony formation ability of ES-2 cells, but inhibition of miR-3187-3p in SK-OV-3 cells led to an inverse trend as well (Fig. 4C). The wound healing and Transwell assays also suggested that the migration (Fig. 4D) and invasion (Fig. 4E) abilities of ES-2 cells, which were initially enhanced by circ_000074, were blocked following further miR-3187-3p upregulation. Again, downregulation of miR-3187-3p restored the aggressiveness of SK-OV-3 cells. In the subsequent sphere formation assay, we observed that the enhancement of oe-circ on the sphere formation ability of ES-2 cells was counteracted by miR mimic, but miR inhibitor was found to recover the stem cell property of SK-OV-3 cells (Fig. 4F).

### miR-3187-3p directly targets ERBB4

**Fig. 5:**
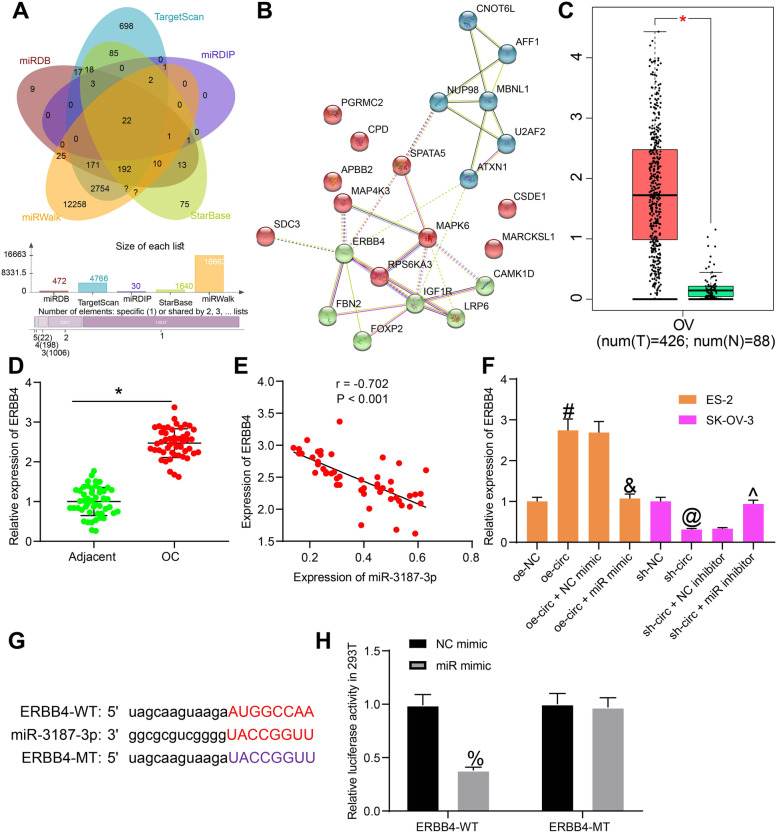
miR-3187-3p directly targets ERBB4. **A**, a Venn diagram for the intersections of the candidate target mRNAs of miR-3187-3p from five bioinformatic systems; **B**, a KEGG pathway enrichment analysis based on the candidate mRNAs; **C**, expression of ERBB4 in OC predicted on the GEPIA database; **D**, mRNA expression of ERBB4 in the collected tissue samples detected by RT-qPCR; **E**, a negative correlation between the expression of miR-3187-3p and ERBB4 mRNA in OC tissues; **F**, mRNA expression of ERBB4 in ES-2 and SK-OV-3 cells after oe-circ/sh-circ/miR-mimic/miR-inhibitor transfection examined by RT-qPCR; **G**, putative binding sequence between miR-3187-3p and ERBB4 3’UTR and the MT sequence for luciferase assay; **H**, luciferase activity of circ-WT and circ-MT in 293 T cells examined by the luciferase reporter gene assay. Data were exhibited as mean ± SD from three repetitions. Differences were analyzed by paired *t* test (**D**), one-way ANOVA (**F**) or two-way ANOVA (**H**); *p < 0.05 vs. adjacent; #*p* < 0.05 vs. oe-NC; &*p* < 0.05 vs. oe-circ +NC mimic; @*p* < 0.05 vs. sh-NC; ^*p* < 0.05 vs. sh-circ + NC inhibitor; %*p* < 0.05 vs. NC mimic; In panel **E**, the correlation between miR-3187-3p and ERBB4 was validated using Pearson’s correlation analysis, *r* = − 0.702, *p* < 0.001

Similarly, we further predicted the target genes of miR-3187-3p using five bioinformatic systems including StarBase, TargetScan (http://www.targetscan.org/vert_72/), miRDB (http://mirdb.org/), miRDIP (http://ophid.utoronto.ca/mirDIP/) and miRwalk (http://mirwalk.umm.uni-heidelberg.de/), and a total of 22 intersected mRNAs were suggested (Fig. 5A). Next, a Kyoto Encyclopedia of Genes and Genomes (KEGG) pathway enrichment analysis was conducted based on these genes, and five crucial genes including ERBB4, RPS6KA3, IGF1R, MAPK6 and LRP6 were identified (Fig. 5B). Then, we searched the expression profiles of these genes in the Gene Expression Profiling Interactive Analysis (GEPIA) database (http://gepia.cancer-pku.cn/index.html). Consequently, only ERBB4 was suggested to be highly expressed in OC patients (Fig. 5C).

Therefore, we examined the expression of ERBB4 in the collected tissue samples using RT-qPCR. The RT-qPCR results suggested that the mRNA expression of ERBB4 was notably higher in the OC tumor tissues than that in the adjacent tissues (Fig. 5D), which showed a negative correlation with miR-3187-3p in OC tissues (Fig. 5E). In ES-2 and SK-OV-3 cells, it was found that the expression of ERBB4 was elevated after circ_0000745 upregulation or miR-3187-3p inhibition, but reduced after circ_0000745 knockdown or miR-3187-3p upregulation (Fig. 5F). Likewise, the putative binding sequence between miR-3187-3p and ERBB4 was obtained from the StarBase system, and the MT sequence was designed as well (Fig. 5G). The subsequent luciferase assay suggested that miR mimic reduced the luciferase activity of ERBB4-WT vector in 293 T cells but it had no effects on the luciferase activity of ERBB4-MT vector (Fig. 5H). This validated the direct binding relationship between miR-3187-3p and ERBB4.

### circ_0000745 sequesters miR-3187-3p and upregulates ERBB4 to mediate the PI3K/AKT signaling pathway in OC cells

**Fig. 6:**
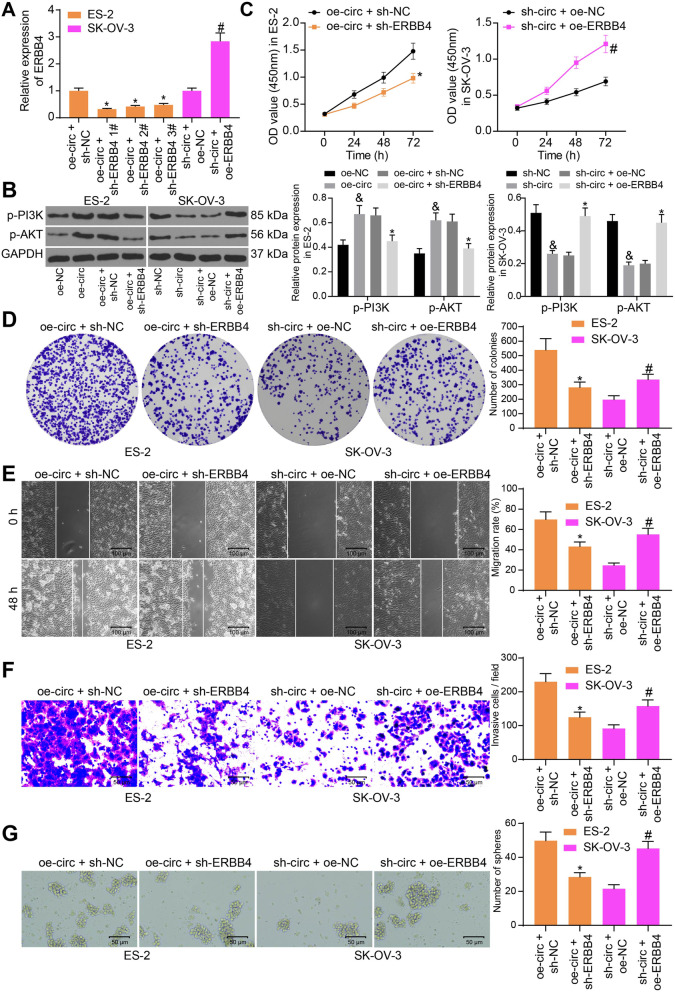
Circ_0000745 upregulates ERBB4 to mediate the PI3K/AKT signaling pathway in OC cells. **A**, transfection efficacy of sh-ERBB4 and oe-ERBB4 in ES-2 or SK-OV-3 cells examined by RT-qPCR; **B**, phosphorylation of AKT and PI3K in ES-2 and SK-OV-3 examined by western blot analysis; **C**, proliferation ability of ES-2 and SK-OV-3 cells examined by the CCK-8 assay; **D**, colony formation ability of ES-2 and SK-OV-3 cells measured by the colony formation assay; **E**, migration ability of ES-2 and SK-OV-3 cells examined by the wound-healing assay; **F**, invasion ability of ES-2 and SK-OV-3 cells determined by the Transwell assay; **G**, stem cell property of ES-2 and SK-OV-3 cells determined by the sphere formation assay. Data were exhibited as mean ± SD from three repetitions. Differences were analyzed by one-way ANOVA (**A**, **D**, **E**, **F** and **G**) or two-way ANOVA (**B** and **C**); *p < 0.05 vs. oe-circ + sh-NC; #*p* < 0.05 vs. sh-circ + oe-NC

Following the findings above, rescue experiments of ERBB4 were conducted as well to validate its relevance to circ_0000745 mediation in OC. Three shRNAs of ERBB4 (sh-ERBB4 1, 2, 3#) were transfected into ES-2 cells after oe-circ transfection, while oe-ERBB4 was transfected into SK-OV-3 cells after sh-circ transfection. The successful transfection was confirmed by RT-qPCR again (Fig. 6A), and the sh-ERBB4 1# with the best interfering efficacy was used for further use.

ERBB4 has been reported as a positive regulator of the PI3K/AKT signaling pathway,[10] while the activation of PI3K/AKT has been reported to promote OC progression [14, 15]. Thus, we speculated that circ_0000745 possible regulates ERBB4 and activates the PI3K/AKT signaling pathway in OC. The western blot assay suggested that the phosphorylation of PI3K and AKT in ES-2 cells was notably enhanced by oe-circ but reduced after sh-ERBB4; whereas the phosphorylation of PI3K and AKT in SK-OV-3 cells was initially suppressed by sh-circ but then restored by oe-ERBB4 (Fig. 6B).

The behaviors of ES-2 and SK-VO-3 cells under these conditions were examined as well. Silencing of ERBB4 reduced the proliferation as well as the colony formation ability of ES-2 cells increased by oe-circ, whereas overexpression of ERBB4 led to inverse trends in SK-OV-3 cells (Fig. 6C-D). The wound healing and Transwell assays also suggested that downregulation of ERBB4 reduced the migratory and invasive potentials of ES-2 cells, though the aggressiveness of SK-OV-3 cells was enhanced upon ERBB4 upregulation (Fig. 6E-F). In addition, the sphere formation ability of ES-2 cells enhanced by oe-circ was blocked by sh-ERBB4. Again, overexpression of ERBB4 enhanced the tumor sphere formation ability of SK-OV-3 cells (Fig. 6G).

### IGF2BP2 binds to circ_0000745 and promotes its expression in OC cells

**Fig. 7:**
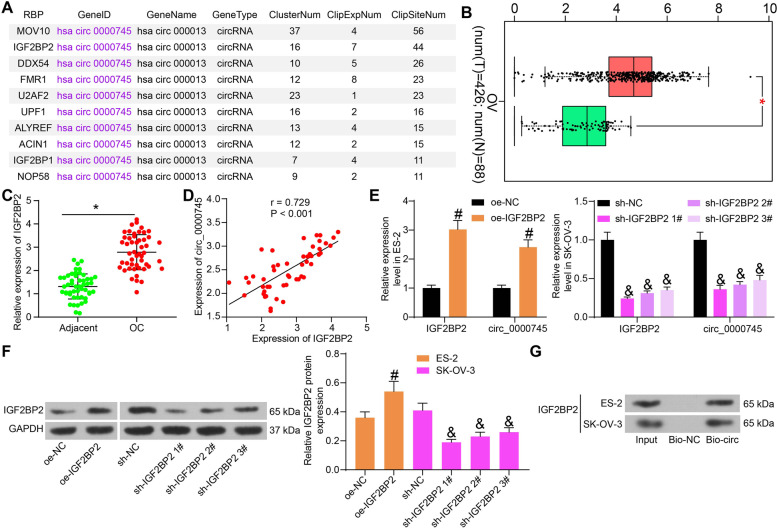
IGF2BP2 binds to circ_0000745 and promotes its expression in OC cells. **A**, candidate RBPs with the potential to regulate circ_0000745 predicted using StarBase; **B**, expression of IGF2BP2 in OC patients predicted on GEPIA; **C**, IGF2BP2 expression in collected OC tissues examined by RT-qPCR; **D**, correlation between the expression of IGF2BP2 and circ_0000745 in OC cells examined by RT-qPCR; **E**, expression of IGF2BP2 and circ_0000745 in ES-2 and SK-OV-3 cells examined by RT-qPCR; **F**, protein level of IGF2BP2 in ES-2 and SK-OV-3 cells after oe-IGF2BP2 or sh-IGF2BP2 transfections determined by western blot analysis;** G**, direct binding relationship between circ_0000745 and IGF2BP2 examined by an RNA pull down assay. Data were exhibited as mean ± SD from three repetitions. Differences were analyzed by paired *t* test (**C**), one-way ANOVA (**F**), or two-way ANOVA (**E**); *p < 0.05 vs. adjacent; @@*p* < 0.01 vs. Bio-NC; #*p* < 0.05 vs. oe-NC; &*p* < 0.05 vs. sh-NC; In panel **D**, the correlation between circ_0000745 and IGF2BP2 was validated using Pearson’s correlation analysis, *r* = − 0.729, *p* < 0.001

The possible upstream regulator responsible for circ_0000745 then aroused our interest. We predicted the RBPs with the potential to regulate circ_000074, and the top 10 RBPs with the highest ‘ClipSiteNum’ score were selected as candidates (Fig. 7A). According the data in GEPIA , only IGF2BP2 showed a notable increase in OC (Fig. 7B). Subsequently, the RT-qPCR results confirmed an upregulation of IGF2BP2 in the OC tumor tissues relative to the adjacent tissues (Fig. 7C), presenting a positive correlation with circ_0000745 (Fig. 7D). To confirm whether IGF2BP2 regulates circ_0000745 expression, oe-IGF2BP2 was transfected into ES-2 cells while sh-IGF2BP2 1, 2, 3# were transfected into SK-OV-3 cells. Next, the expression of IGF2BP2 and circ_0000745 in cells was notably enhanced. RT-qPCR found that oe-IGF2BP2 increased the expression of IGF2BP2 and the abundancy of circ_0000745 in ES-2 cells, whereas sh-IGF2BP2 1, 2, 3# suppressed the expression of IGF2BP2 and circ_0000745 in SK-OV-3 cells (Fig. 7E). In line, the western blot analysis suggested that oe-IFG2BP2 significantly promoted the protein level of IFG2BP2 ES-2 cells, and sh-IGF2BP2 1, 2, 3# suppressed the IFG2BP2 protein in SK-OV-3 cells (Fig. 7F). The sh-IGF2BP2 1# with the best interfering silencing efficacy was selected for subsequent use. The binding relationship between IGF2BP2 and circ_0000745 was validated through an RNA pull-down assay. Compared to Bio-NC, an enrichment of IGF2BP2 protein was found in the complexes pulled down by Bio-circ (Fig. 7G).

### Silencing of IGF2BP2 suppresses the tumorigenesis of OC cells *in vivo*

**Fig. 8:**
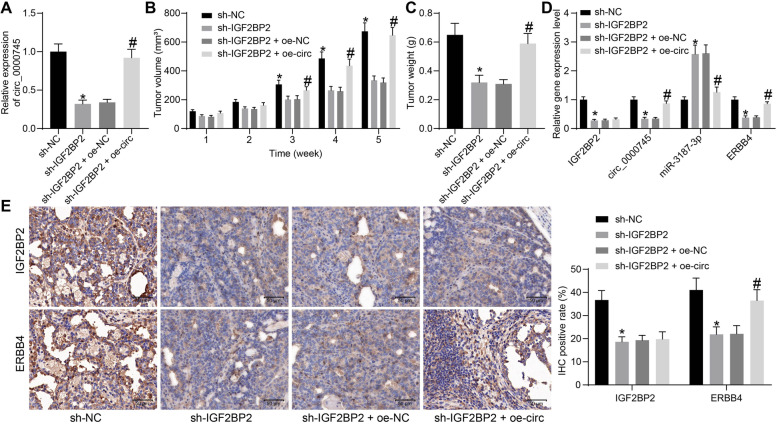
Silencing of IGF2BP2 suppresses the tumorigenesis of OC cells in vivo. **A**, expression of circ_0000745 in SK-OV-3 cells examined by RT-qPCR; **B**, growth rate of xenograft tumors; **C**, weight of the xenograft tumors on the 35th d; **D**, expression of IGF2BP2 mRNA, circ_0000745, miR-3187-3p, and ERBB4 mRNA in tumor tissues examined by RT-qPCR; **E**, protein levels of IGF2BP2 and ERBB4 tumor tissues determined by western blot analysis. In each group, *n* = 5; the mean value was calculated. Data were exhibited as mean ± SD. Differences were analyzed by the two-way ANOVA (**A** and **C**) or two-way ANOVA (**B**, **D** and **E**); *p < 0.05 vs. sh-NC; #*p* < 0.05 vs. sh-IGF2BP2 + oe-NC

SK-OV-3 cells stably transfected with sh-IGF2BP2 were further transfected with oe-circ or the oe-NC. The transfection of oe-circ restored the expression of circ_0000745 in cells (Fig. 8A). The cells stably transfected with sh-NC, sh-IGF2BP2, sh-IGF2BP2 + oe-NC and sh-IGF2BP2 + oe-circ were injected into nude mice for animal experiments.

It was observed that the growth rate of xenograft tumors and the tumor weight on the 35th d were significantly suppressed by IGF2BP2 silencing but restored after circ_0000745 overexpression (Fig. 8B-C). The gene expression in tumor tissues was then examined. The RT-qPCR results indicated that silencing of IGF2BP2 led to a decline in the expression of circ_0000745 and ERBB4 but an increase in the expression miR-3187-3p. Overexpression of circ_0000745 did not significantly affect the expression of IGF2BP2, but it suppressed the expression of miR-3187-3p and restored the level of ERBB4 (Fig. 8D) Likewise, the IHC results indicated that in the sh-IGF2BP2 group, the protein levels of IGF2BP2 and ERBB4 in tissues were significantly reduced, but the protein level of ERBB4 was enhanced after circ_0000745 restoration (Fig. 8E).

## Discussion

The late presentation and highly malignancy make OC as a serious health concern among women, which demands novel understanding and insights in the pathophysiology of the disease as well as the development of new therapeutic options. CircRNAs have been increasingly recognized as biomarkers for OC diagnosis and prognosis as well as therapeutic targets for OC [16, 17]. In this study, we reported that circ_0000745 can be upregulated by an RBP IGF2BP2 and promotes aggressiveness and stemness of OC cells through a miR-3187-3p/ERBB4/PI3K/AKT axis.

Emerging evidence has reported the versatile functions of circRNAs in OC progression [18, 19]. Circ_0000745 has been suggested as a potential diagnostic biomarker for gastric cancer [20]. In addition, upregulation of circ_0000745 was found to induce proliferation of cells in acute lymphoblastic leukemia [21]. The oncogenic role of circ_0000745 has been reported in another major gynecologic tumor cervical cancer by triggering cell proliferation, migration, invasion and EMT [7, 22]. Here, our study first confirmed high expression of circ_0000745 in the collected OC tissues and the acquired OC cell lines. After confirmation of the high stability of circ_0000745 using RNase R treatment, altered expression of circ_0000745 was introduced in OC cell lines. The cellular experiments suggested that downregulation of circ_0000745 reduced proliferation, EMT and metastasis, as well as the stem cell properties of the OC cells. Therefore, we reported that circ_0000745 also functions as an oncogene in OC like the functions reported in other malignancies. The promoting effect of circ_000745 on stemness of OC cells also suggested that targeting circ_000745 may help overcome drug resistance in OC cells.

CircRNAs frequently serve as sponges for miRNAs to regulate the expression of downstream mRNAs. Here, after identification of a cytoplasm-localization of circ_0000745 in OC cells, we explored the target transcripts of circ_0000745 using several bioinformatic systems with miR-3187-3p confirmed as a promising target. Though there is limiting evidence concerning the function of miR-3187-3p in OC before, this miRNA has been reported as a non-invasive biomarker, indicating favorable prognosis in patients with bladder cancer [9]. Importantly, we confirmed poor expression of miR-3187-3p in the collected tumor tissues, and the rescue experiments suggested that miR-3187-3p upregulation blocked the functions of circ_0000745 overexpression and suppressed the malignant behaviors of cells, indicating that downregulation of miR-3187-3p was implicated in the oncogenic roles of circ_0000745 in OC. Likewise, using integrated bioinformatic analyses, we confirmed ERBB4 as the target of miR-3187-3p. ERBB4 is an important member of EGFR family whose overexpression and activation has been suggested to be associated with advanced tumor states and poor patient outcomes [23]. ERBB4 activates the related genes in nucleus and enhances cell division and proliferation [24]. Interestingly, though the EGFR family is closely linked to cell proliferation and oncogenic events, ERBB4 has been confirmed as an exception in some cases [10]. The ERBB4 abundancy was predicted to be decreased in several cancers, including lung, esophageal, cervical, and bladder carcinoma; however, the ERBB4 expression in OC was suggested to remain high [10]. Also, high incidence of ERBB4 has been found in 98% of the 202 OC cases in a study by Davie et al. [25]. In addition, high expression of ERBB4 was reported to be linked to increased resistance of OC to platinum-based treatment [26]. Here, we confirmed that ERBB4 was highly expressed in the collected OC tissues with a positive correlation with circ_0000745. Further experiments confirmed that downregulation of ERBB4 blocked the oncogenic function of circ_0000745 in OC cells while upregulation of ERBB4 promoted the proliferation, metastasis and stemness of OC cells. ERBB4 has been reported as an important regulator of the PI3K/AKT signaling pathway to enhance the stem cell activity, angiogenesis, and proliferation of cancer cells [10, 24, 27]. The PI3K/AKT signaling pathway plays critical roles in cell survival, proliferation and growth, and deregulation of this pathway is frequently involved in cancer progression, including OC [28]. Targeting PI3K was also suggested as a potential therapeutic option of OC management [10]. We therefore speculated that there is a similar ERBB4/PI3K/AKT axis in OC. Consequently, overexpression of circ_0000745 in this study was found to enhance the PI3K/AKT activation, while this enhancement was blocked upon ERBB4 inhibition.

RBPs are a major type of proteins with the capability of binding to different RNA transcripts. For instance, an RBP FUS was found to bind to circ_002136 and govern the angiogenesis in glioma [29]. Likewise, an RBP U2AF2 was reported to bind to and enhance the stability and abundancy of circRNA ARF1 [30]. Here, our bioinformatic analyses suggested IGF2BP2 as a candidate RBP binding to circ_0000745. IGF2BP2 has been suggested as one of the major RBPs which helped form the upper stream regulatory elements of several hub circRNAs in triple-negative breast cancers [31]. Here, we confirmed that IGF2BP2 was highly expressed in the collected OC tissues that showed a positive correlation with circ_0000735. Therefore, the upregulation of circ_0000735 in OC is possibly, at least partially, regulated by IGF2BP2.

## Conclusions

In conclusion, our study reported that the RBP IGF2BP2 enhances circ_0000745 abundancy. The circ_0000745 further promotes proliferation, EMT, and stemness of OC cells by sequestering miR-3187-3p and activating the subsequent ERBB4/PI3K/AKT axis. We hope these findings may offer novel insights in the pathogenesis of OC. Targeting circ_0000745 may serve as a novel option for the OC management.

## Data Availability

The datasets during and/or analysed during the current study available from the corresponding author on reasonable request.

## Abbreviations

ANOVA: Analysis of variance
EGFR: Epidermal growth factor receptor
ERBB4: Erb-b2 receptor tyrosine kinase 4
FBS: Fetal bovine serum
GAPDH: Glyceraldehyde-3-phosphate dehydrogenase
HRP: Horseradish peroxidase
IGF2BP2: Insulin like growth factor 2 mRNA binding protein 2
IgG: Immunoglobulin G
OC: Ovarian cancer
OD: Optical density
PBS: Phosphate-buffered saline
RBPs: RNA binding proteins
SPECC1: Sperm antigen with calponin homology and coiled-coil domains 1
3’UTR: 3’untranslated region
